# Molecular Subtype Identification and Prognostic Prediction of Pancreatic Cancer Based on m6A/m5C/m1A‐Related Genes

**DOI:** 10.1111/jcmm.71251

**Published:** 2026-06-28

**Authors:** Yunyang Wang, Xueqing Hong, Dingge Cao, Mingzhen Wang, Abulaihaiti Tuergong, Hongrui Liu, Yuxuan Guo, Xia Ting, Xujun Liu, Xiao Huo, Wenzhe Si

**Affiliations:** ^1^ Department of Laboratory Medicine, State Key Laboratory of Vascular Homeostasis and Remodeling, Key Laboratory of Cardiovascular Molecular Biology and Regulatory Peptides Peking University Third Hospital Beijing China; ^2^ Department of Pathology, School of Basic Medical Sciences Peking University Third Hospital, Peking University Health Science Center Beijing China; ^3^ Department of Laboratory Medicine Peking University First Hospital Beijing China; ^4^ Center of Basic Medical Research Peking University Third Hospital Beijing China

**Keywords:** molecular subtype, pancreatic cancer, prognostic prediction, RNA methylation, TRMT61B

## Abstract

Pancreatic cancer, characterized by an unfavourable prognosis, necessitates early diagnosis and prompt therapeutic intervention. This study aimed to delineate the functional implications of m6A/m1A/m5C‐related genes in pancreatic carcinogenesis and establish a prognostic model. Utilizing data from the TCGA‐PAAD cohort (n=178) combined with GTEx normal pancreatic tissues (n=332), we identified 43 genes associated with m^6^A/m^1^A/m^5^C methylation pathways. Significant expression differences in these genes were observed between neoplastic and non‐neoplastic tissues, correlating with multiple biological pathways. Consensus clustering divided pancreatic cancer patients into two distinct molecular subtypes exhibiting pronounced survival differences. A prognostic risk‐scoring model was developed based on nine m^6^A/m^1^A/m^5^C‐related genes, demonstrating efficacy in predicting patient outcomes. We constructed a nomogram integrating clinical variables with the risk score to enhance prognostic precision. Among these genes, a comprehensive investigation was conducted using immunohistochemistry and multicolor immunofluorescence to elucidate that higher TRMT61B expression in tumour tissues is significantly associated with higher pancreatic cancer stage, and might relate to an immunosuppressive and dysfunctional tumour immune microenvironment. This study reveals the role of m^6^A/m^1^A/m^5^C associated genes, especially TRMT61B, in the epigenetic regulation of pancreatic cancer, providing evidence supporting their potential as novel prognostic biomarkers.

## 1 Introduction

Pancreatic cancer, predominantly pancreatic ductal adenocarcinoma, is a highly lethal malignancy with an overall 5‐year survival rate of approximately 10% [1]. Early‐stage surgical resection remains the only potentially curative treatment, highlighting the critical need for early diagnosis and timely intervention [2]. Consequently, developing robust prognostic indicators is essential to improve patient outcomes.

N^6^‐methyladenosine (m^6^A), N^1^‐methyladenosine (m^1^A), and 5‐methylcytosine (m^5^C) modifications play pivotal roles in eukaryotic mRNA regulation [3, 4, 5]. These reversible modifications influence nearly every aspect of RNA metabolism, including stability, splicing, export, and translation, thereby exerting profound effects on gene expression. Their dysregulation is increasingly implicated in various pathological states, particularly in cancer, where they can act as critical drivers of tumour initiation, progression, and metastasis [6]. For instance, prognostic models based on m^6^A/m^5^C/m^1^A‐related genes have been established for cancers like cervical cancer, myeloid leukaemia, and thyroid cancer, linking them to survival and immune infiltration [7, 8, 9]. However, the specific roles of these regulatory genes in pancreatic cancer progression remain incompletely understood.

Exploring the mechanisms of m^6^A, m^5^C, and m^1^A methylation‐modulated genes in pancreatic cancer prognosis and treatment is therefore vital. We utilized data from The Cancer Genome Atlas (TCGA) to investigate the biological functions, interaction networks, and prognostic significance of m^6^A/m^1^A/m^5^C regulatory genes in pancreatic cancer (PAAD). Based on these genes, we further employed LASSO regression to construct a risk model and nomogram for predicting PAAD patient prognosis.

Notably, we identified TRMT61B as playing an epigenetic regulatory role in pancreatic cancer. TRMT61B, which is a mitochondrial RNA methyltransferase, has been recognized as a potential biomarker and therapeutic target in cancer [10]. While TRMT61B has been implicated in hepatoblastoma, nephroblastoma, and oral squamous cell carcinoma [10, 11, 12, 13], its function in pancreatic cancer is unclear. Building on the exploration of m^6^A/m^5^C/m^1^A‐associated genes, this study investigates the expression profile and potential regulatory mechanisms of TRMT61B in pancreatic cancer. Our aim is to clarify its impact on tumorigenesis and progression, as well as its diagnostic and prognostic value.

## 2 Materials and Methods

### 2.1 Collection of PAAD Dataset

PAAD samples with clinical and survival information were collected from TCGA_PAAD, GSE62452 (GEO), GSE57495 (GEO), and ICGC_PACA_AU cohorts. Normal pancreatic tissue data (*n* = 328) were obtained from GTEx. TPM values for TCGA and ICGC_PACA_AU were acquired from the GDC portal (https://portal.gdc.cancer.gov/) [14]. GEO datasets (GSE62452, GSE57495) were downloaded from NCBI GEO (http://www.ncbi.nlm.nih.gov/geo/).

### 2.2 Identification of m6A/m1A/m5C‐Related Gene Expression and Variation Levels

Differentially expressed genes (DEGs) between 178 PAAD tumours and 332 normal tissues (TCGA‐PAAD + GTEx) were screened using the “limma” R package (adj *p* < 0.05 and |log2FC| > 2). Somatic mutations in m^6^A/m^1^A/m^5^C‐related genes were analysed using “maftools”. Copy number variation (CNV) analysis was performed; CNV > 0.2 were considered “gains” and < −0.2 as “losses”.

### 2.3 Analysis of m6A/m1A/m5C Methylation Regulation

By collating m^6^A/m^1^A/m^5^C methylation regulatory genes from previous studies, we initially identified 45 m^6^A/m^1^A/m^5^C‐related genes [15]. VIRMA was excluded due to its absence in GTEx, and RBMY1A1 was excluded because its expression was zero for all samples in the TCGA dataset, resulting in a final set of 43 genes. Expression differences between tumour and normal tissues were analysed. Protein–protein interaction (PPI) networks were investigated using the STRING database [16]. Co‐expression and correlation among the 43 genes were assessed.

### 2.4 Construction of the Risk Scoring Model

LASSO Cox regression with 1000‐fold cross‐validation was used to identify the optimal prognostic gene set and their regression coefficients (β) [17]. The risk score was calculated as follows:

$$\text{Risk score}=\sum_{i=1}^{n}\;\text{Gene}\;\text{expri}\times \mathrm{\beta i}$$

where *n* is the number of genes, Gene expri is the expression of gene i, and *β*i is its coefficient. Patients were stratified into high‐risk and low‐risk groups based on the median risk score. Model performance was evaluated using ROC curves (AUC) via the “timeROC” package. Kaplan–Meier survival analysis with log‐rank tests compared OS between groups. Univariate and multivariate Cox regression assessed the risk score and clinical features as independent prognostic factors. TCGA served as the training set; GEO and ICGC datasets were validation sets.

### 2.5 Construction and Evaluation of the Nomogram Survival Model

A nomogram survival model is a graphical calculating scale that predicts an individual's probability of survival over a specific time period by integrating multiple rognostic factors [18]. A prognostic nomogram integrating clinical features and the risk score was built using multivariate Cox regression (“regplot” package). Calibration plots and Decision Curve Analysis (DCA) evaluated its efficacy.

### 2.6 Consensus Clustering Analysis

Consensus clustering analysis is a robust, resampling‐based method used to determine the optimal number of clusters and assess the stability of classifications within a dataset [19]. Based on the model genes, we performed consensus clustering (CC) using the “ConsensusClusterPlus” package to identify subtypes of PAAD. The CC parameter “maxK” was set to “10”, “clusterAlg” was set to “pam”, and “distance” was set to “pearson”.

### 2.7 Functional Enrichment Analysis

The “clusterProfiler” R package was used to identify potential biological pathways based on DEGs.

### 2.8 Immune Cell Infiltration Analysis and Anti‐Tumour Immune Response Analysis

CIBERSORT estimated tumour‐infiltrating immune cell composition. Wilcoxon tests compared immune cell components, T‐cell exhaustion genes, antigen presentation genes, interferon activity genes, cytolytic activity genes, integrin genes, and kinase genes between subtypes and risk groups.

### 2.9 Immunohistochemistry Analysis

Immunohistochemical (IHC) analysis was performed using a tissue microarray containing 150 samples (OUTDO BIOTECH, Shanghai, China; catalogue number: HPanA150CS02), including 78 pancreatic ductal adenocarcinoma (PDAC) samples and 72 adjacent non‐tumour tissue samples. IHC staining was performed by Servicebio Technology (Wuhan, China) using the corresponding antibodies.

Tissue sections were deparaffinized, rehydrated, and subjected to antigen retrieval and treatment with 3% hydrogen peroxide. Regions of interest were demarcated with a hydrophobic pen, and non‐specific binding sites were blocked with serum. The sections were then incubated with TRMT61B primary antibody (Immunoway, San Jose, CA, USA, catalog number: YN6645) overnight at 4°C. Following primary antibody incubation, sections were incubated with polymer HRP goat anti rabbit IgG(H+L) secondary antibody (Immunoway) at room temperature. This was followed by chromogenic development, haematoxylin counterstaining, and a final series of dehydration, clearing, and mounting steps (Abcam, Cambridge, UK). Positive signals were quantified using ImageJ software, expressed as the percentage of positive cells per field. Data are presented as mean ± standard deviation (SD) and were compared using Student's *t*‐test, with a *p* < 0.05 considered statistically significant.

### 2.10 Multicolor Immunofluorescence Assay

To identify various cell subsets within the tumour microenvironment (TME) in pancreatic cancer tissues, multiplex immunofluorescence staining was carried out on a tissue microarray (OUTDO BIOTECH, Shanghai, China) Multiplex staining procedures were performed using the PANO 7‐plex IHC kit (Panovue, Beijing, China; catalogue number: 0004100100). The procedure involved the sequential application of different primary antibodies. Each application was followed by incubation with polymer HRP goat anti mouse/rabbit IgG(H+L) secondary antibody (Immunoway) and tyramide signal amplification (TSA). A microwave treatment step was incorporated after each TSA cycle to strip the antibodies. Finally, after all human antigens were labelled, the nuclei were counterstained with 4′,6‐diamidino‐2‐phenylindole (DAPI). Positive signals were quantified using “QuPath‐0.6.0” software, expressed as the percentage of positive cells per tissue. The quantitative association between TRMT61B expression and infiltration levels of various immune cells was analysed using Pearson correlation analysis and simple linear regression analysis. *p* < 0.05 was considered statistically significant.

### 2.11 Cell Culture

MIA‐PaCa 2 and ASPC‐1 cells were cultured in Dulbecco’s modified Eagle’s medium (DMEM) or Roswell Park Memorial Institute (RPMI) 1640 medium supplemented with 10% (vol/vol) fetal bovine serum (FBS) (Wuhan Procell Biotechnology Co. Ltd. Wuhan, China), 1% (vol/vol) penicillin‐streptomycin. Cells were passaged at 80%–90% confluency. After removing the medium and washing with PBS, cells were detached using 0.25% trypsin–EDTA at 37°C for 2–3 min. Digestion was stopped with complete medium. The cell suspension was centrifuged (1000 rpm, 5 min), resuspended, and seeded at a split ratio of 1:3 in fresh medium. Cells were cultured at 37°C with 5% CO_2_. Cells were cryopreserved using serum‐free cell cryopreservation medium (CELLSAVING) (New Cell & Molecular Biotech, Suzhou, China, catalog number: C40100).

### 2.12 Lentiviral Transduction

Stable cell lines were generated by TRMT61B‐KO lentiviral (Corues Biotechnology, Nanjing, China) transduction. Cells were seeded in 6‐well plates (20%–30% confluency) and transduced the next day by adding polybrene (8 µg/mL) and lentivirus (MOI = 10) in 1 mL fresh medium per well. After 16–24 h incubation, the medium was replaced. Transduction efficiency was evaluated ~72 h post‐infection via fluorescence microscopy. Selection was performed using 1.5 μg/mL puromycin.

### 2.13 Colony‐Formation Assay

The colony‐forming activity was assessed as previously described. Briefly, 5000 cells were seeded and cultured for 7 days. Colonies were then fixed with 4% paraformaldehyde for 10 min and stained with 0.1% crystal violet for 10 min. After washing and air‐drying, colonies were photographed and counted for quantitative analysis.

### 2.14 Cell Proliferation Assay

Seed 4000 cells per well in a 96‐well plate. Use the IncuCyte live‐cell analysis system to automatically capture microscopic images at the same location every 3 h to monitor cell proliferation, and culture the cells for 72 h. Assess proliferation based on cell confluence, normalized to the value at 0 h as calculated by the IncuCyte software.

### 2.15 Transwell Invasion Assay

A Transwell invasion assay was performed to evaluate cell invasive ability. Briefly, cells were trypsinized, centrifuged, and resuspended in serum‐free medium. For each 24‐well plate Transwell insert, 200 μL of serum‐free cell suspension containing 1 × 10^5^ cells was seeded into the upper chamber. The lower chamber was filled with 600 μL of complete medium containing 20% FBS as a chemoattractant. After 48 h of incubation, the invaded cells on the lower surface were fixed with 600 μL of 4% paraformaldehyde for 30 min, washed with PBS, and stained with 600 μL of crystal violet for 10 min. After washing and air drying, the membrane was imaged under an inverted microscope. Invaded cells in 20× magnification fields were counted and quantitatively analysed using ImageJ software. Results were expressed as the number of migrated cells per field.

### 2.16 Statistical Analysis

All analyses used R software (v4.1.1). Wilcoxon rank‐sum test compared two groups; Kolmogorov–Smirnov test compared multiple groups. Survival differences were assessed by log‐rank test. *p* < 0.05 was considered significant.

## 3 Results

### 3.1 Gene Screening and Correlation Analysis

**FIGURE 1:**
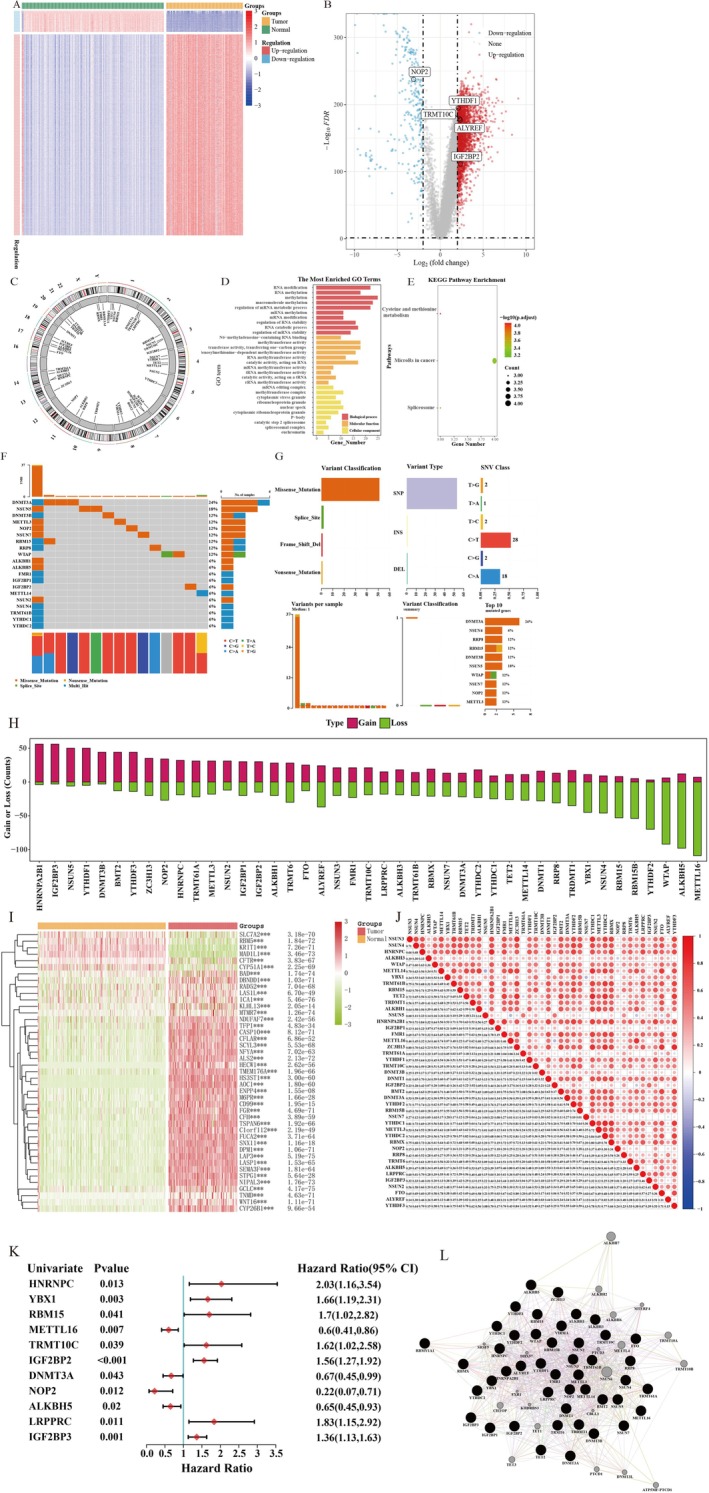
Differential genes in PAAD patients, Landscape of Variations in PAAD Patients and Analysis of m6A/m1A/m5C‐related genes. (A) Heatmap of DEGs between PAAD and normal tissues. (B) Volcano plot of DEGs between PAAD and normal tissues (Blue: Downregulated DEGs; Red: Upregulated DEGs; Grey: Unchanged genes). (C) Chromosomal location and expression of m6A/m1A/m5C‐related genes in the TCGA cohort. (D) GO enrichment analysis based on m6A/m1A/m5C‐related genes. (E) KEGG enrichment analysis based on m6A/m1A/m5C‐related genes. (F, G) Tumour SNV analysis of m6A/m1A/m5C‐related genes in the TCGA cohort. (H) CNV values of m6A/m1A/m5C‐related genes in the TCGA cohort. (I) Expression of 43 m6A/m1A/m5C regulatory genes in tumour and normal tissues; (J) Heatmap of correlation analysis for m6A/m1A/m5C gene expression; (K) Forest plot showing the relationship between 11 m6A/m1A/m5C‐related genes and OS prognosis; (L) Protein–protein interaction network among m6A/m1A/m5C regulatory genes.

We identified 3133 DEGs between PAAD and normal tissues (TCGA‐PAAD + GTEx): 2836 upregulated and 297 downregulated (Figure 1A,B). Analysis of 43 m^6^A/m^1^A/m^5^C‐related genes revealed their chromosomal locations and expression patterns (Figure 1C). KEGG and GO enrichment indicated involvement in RNA modification, methylation, and metabolic pathways (Figure 1D,E). Somatic mutation analysis showed missense mutations predominated; DNMT3A had the highest mutation frequency (24%) (Figure 1F,G). CNV analysis revealed frequent gains and losses across these genes (Figure 1H). Significant expression differences were observed between PAAD and normal tissues for the 43 genes (Figure 1I). A significant correlation was observed between co‐expression patterns (Figure 1J) and protein–protein interactions (Figure 1L). Prognostic analysis linked 11 genes to Overall Survival (OS) (*p* < 0.05): METTL16, DNMT3A, NOP2, and ALKBH5 were protective factors; seven others were risk factors (Figure 1K).

### 3.2 Unsupervised Clustering Results and Validation of m6A/m1A/m5C‐Related Model Genes

**FIGURE 2:**
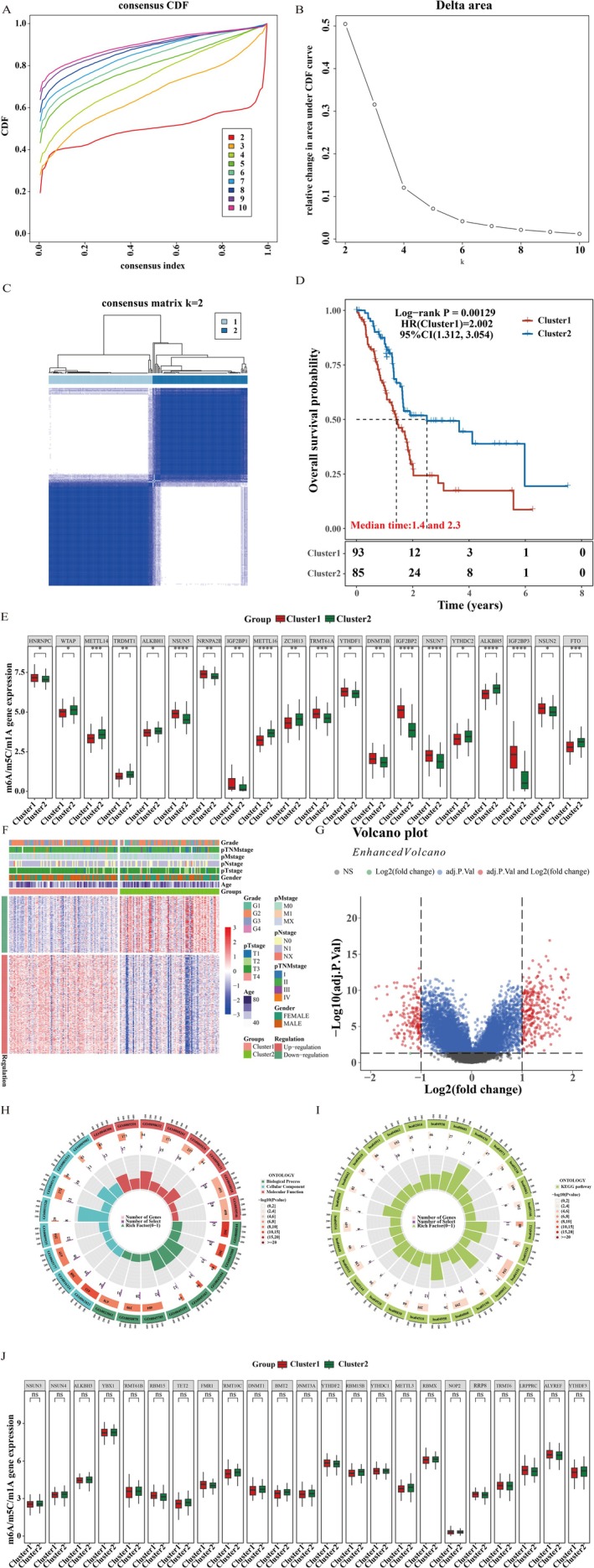
Unsupervised clustering of m6A/m1A/m5C‐related genes. (A, B) Empirical cumulative distribution function plots showing the consensus distribution for each *k* value (from 2 to 10). (C) When *k* = 2, PAAD patients are divided into two molecular clusters based on the m6A/m1A/m5C‐related gene profile. (D) Kaplan–Meier analysis of the prognosis for PAAD patients belonging to two different molecular clusters. (E) Significant expression distribution of 20 m6A/m1A/m5C‐related genes in the two subgroups. (F) Heatmap of differentially expressed genes in the two subtypes. (G) Volcano plot of differentially expressed genes in the two subtypes. (H) GO enrichment results for 476 subtype‐differential genes. (I) KEGG functional enrichment results for 476 subtype‐differential genes. (J) Expression distribution of 23 non‐significant m6A/m1A/m5C‐related genes in the two subgroups.

To explore and identify PAAD subtypes, we performed consensus clustering analysis using 43 m^6^A/m^1^A/m^5^C‐related genes. The most distinct differences between subgroups were observed when *k* = 2, indicating that the 178 PAAD patients could be well divided into two clusters (Figure 2A–C). There was a significant difference in overall survival (OS) times between the two clusters (*p* = 0.00129, Figure 2D). Cluster 2 was associated with better prognosis, while Cluster 1 was associated with poor prognosis. We then further observed the expression distribution of m^6^A/m^1^A/m^5^C‐related genes in the two subtypes (Figure 2E and Figure 2J). Analysis identified 476 DEGs between subtypes: 303 upregulated in Cluster 1, 173 downregulated (Figure 2F,G). GO and KEGG enrichment of these DEGs revealed subtype‐specific pathway associations (Figure 2H,I). Validation of Subtype Classification in External Datasets was shown in Figure S1. To validate the results of PAAD subtypes, we performed consistent clustering analysis using the same 43 m^6^A/m^1^A/m^5^C‐related genes on three additional external datasets. Our analysis of the ICGC_PACA_AU dataset revealed that the most pronounced differences between subgroups were observed when *k* = 2, indicating that the 267 PAAD patients could be well divided into two clusters (Figure S1A). There was a significant difference in OS prognosis between the two clusters (*p* = 0.0192, Figure S1B), with cluster 2 being associated with a good prognosis and cluster 1 being associated with a poor prognosis. Analysing the GSE62452 dataset, we again found that the most pronounced differences between subgroups were observed when *k* = 2, suggesting that the 66 PAAD patients could be well divided into two clusters (Figure S1C). There was a significant difference in OS prognosis between the two clusters (*p* = 0.0242, Figure S1D), with cluster 2 being associated with a good prognosis and cluster 1 being associated with a poor prognosis. Finally, analysing the GSE57495 dataset, we once more found that the most pronounced differences between subgroups were observed when *k* = 2, indicating that the 63 PAAD patients could be well divided into two clusters (Figure S1E). There was a significant difference in OS prognosis between the two clusters (*p* = 0.0307, Figure S1F), with cluster 2 again being associated with a good prognosis and cluster 1 being associated with a poor prognosis. Through the analysis of these three external datasets, we found that the conclusions were largely consistent with the results of the PAAD subtype analysis from the TCGA dataset, thus validating the molecular subtype results.

### 3.3 Construction and Validation of the Prognostic Model

**FIGURE 3:**
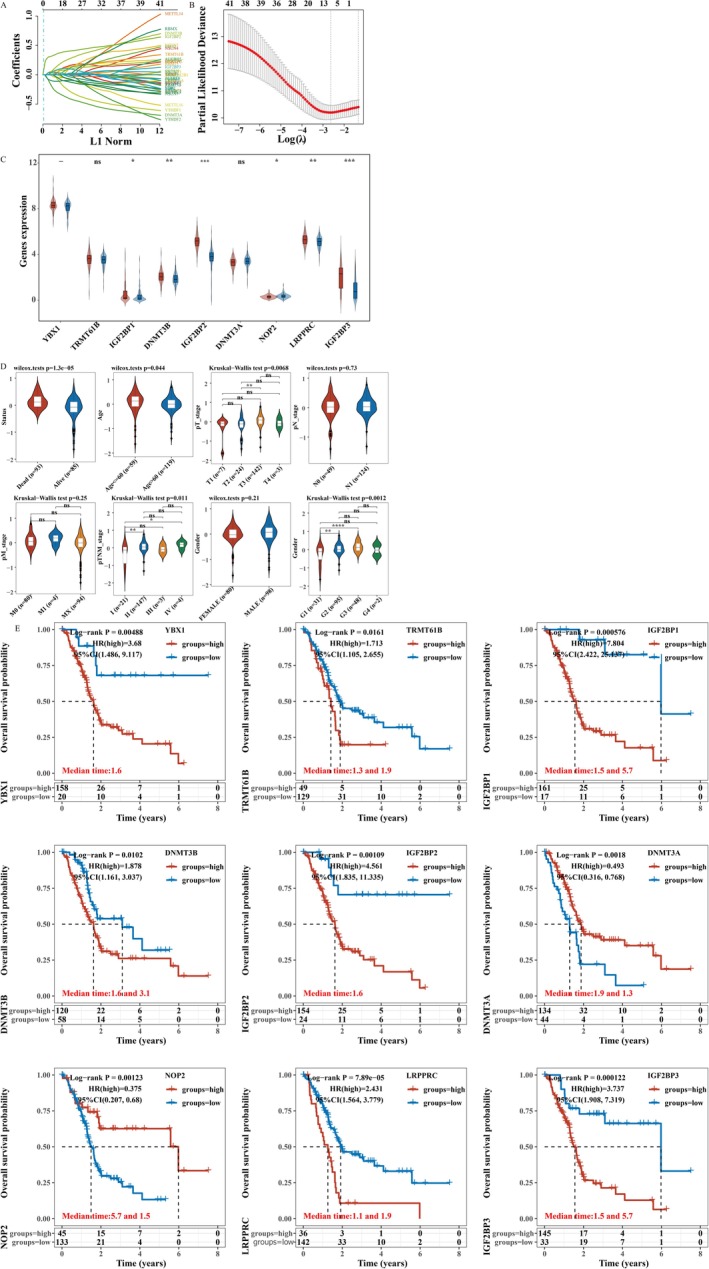
(A, B) LASSO variable selection process, with confidence intervals at each lambda and the trajectory of each independent variable. The horizontal axis represents the log value of the independent variable lambda, and the vertical axis represents the coefficient of the independent variable; (C) Expression distribution of the 9 model genes in different risk groups; (D) Relationship between the risk model and clinical characteristics. (E) Distribution of the Kaplan–Meier (KM) curves for genes in the model.

Firstly, we used LASSO regression to construct a prognostic model based on 43 m6A/m1A/m5C‐related genes (Figure 3A,B), retaining the genes with the smallest result in the model, which are the 9 genes at lambda.1se = 0.07206146 as the final prognostic model. The KM curves for the 9 genes are shown in Figure 3E, and the model formula is as follows:

$$\begin{array}{c} {\text{Methyscore}}_{9}=0.0459^{*}\mathrm{ex}p^{\mathrm{YBX}1}+0.01803^{*}\mathrm{ex}p^{\text{TRMT}61B}+0.000196^{*}\mathrm{ex}p^{\mathrm{IGF}2\mathrm{BP}1}\\ +0.0231^{*}\mathrm{ex}p^{\text{DNMT}3B}+0.3070^{*}\mathrm{ex}p^{\mathrm{IGF}2\mathrm{BP}2}-0.1261^{*}\mathrm{ex}p^{\text{DNMT}3A}-0.0881^{*}\mathrm{ex}p^{\mathrm{NOP}2}\\ +0.0159^{*}\mathrm{ex}p^{\text{LRPPRC}}+0.0384^{*}\mathrm{ex}p^{\mathrm{IGF}2\mathrm{BP}3} \end{array}$$

Next, we analysed the expression of these 9 model genes in different risk groups, with the results shown in Figure 3C. We further analysed Methyscore and the distribution of clinical characteristics and found that Methyscore showed significant differences in survival status, age group, T stage, Grade, and TNM Stage, as shown in Figure 3D.

**FIGURE 4:**
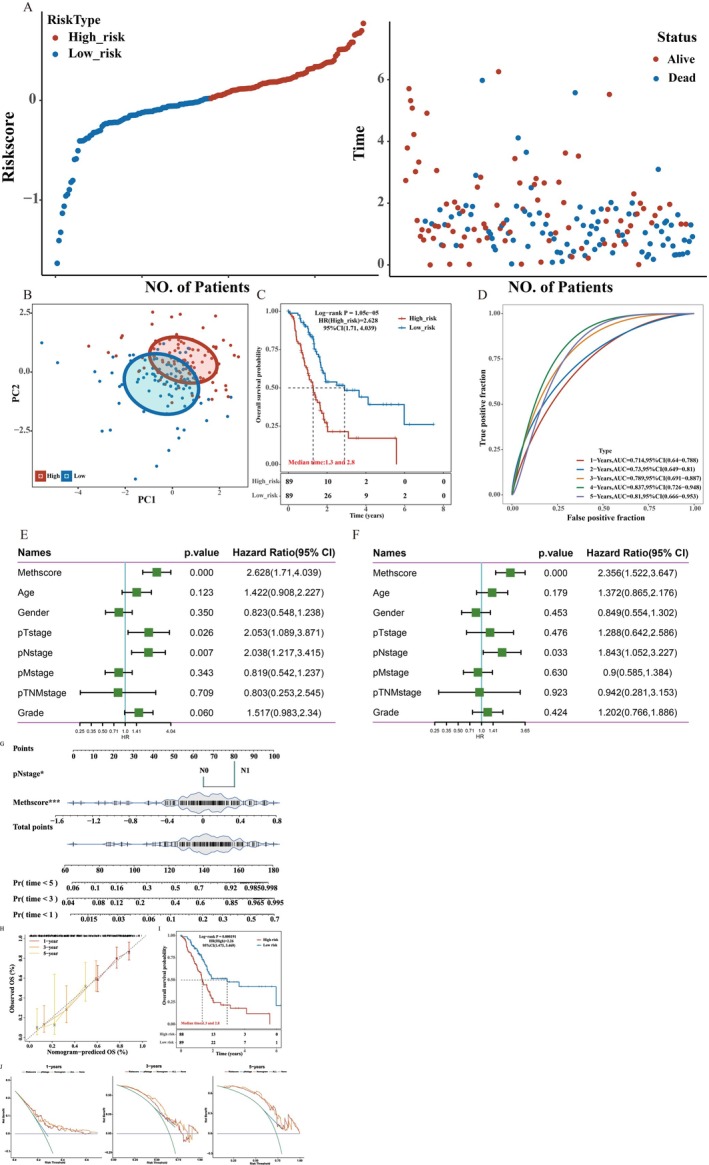
Training Set for the Gene Signature Prediction Model and Establishment and Evaluation of the Nomogram Survival Model. (A) Distribution of Methyscore adjusted for survival status and time in the TCGA cohorts (Left and right plots share the same x‐axis corresponding to NO. of patients); (B) Principal Component Analysis (PCA) plot based on Methyscore in the TCGA cohorts; (C) Overall survival of patients with low and high Methyscore in the TCGA cohorts; (D) ROC curves and AUC values in the TCGA cohorts. (E) Univariate Cox regression analysis of the risk model and clinical parameters; (F) Multivariate Cox regression analysis of the risk model and clinical parameters; (G) Development of a prognostic nomogram to predict 1‐year, 3‐year, and 5‐year OS for patients in the training set; (H) Calibration curve to assess the agreement between predicted and actual OS; (I) Nomogram KM survival curve; (J) DCA to evaluate the clinical decision‐making benefit of the nomogram.

Risk scores were calculated for TCGA (training) and external datasets (GSE62452, GSE57495, ICGC_PACA_AU) (Figure 4A, S2A). Figure S2A showed distribution of Methyscore adjusted for survival status and time in the ICGC_PACA_AU, GSE62452, and GSE57495 cohorts. PCA confirmed separation based on the 9‐gene signature (Figure 4B, S2B). Figure S2B showed Principal Component Analysis (PCA) plot based on Methyscore in the ICGC PACA AU.GSE62452, and GSE57495 cohorts. Patients with high Methyscore had significantly worse OS in all cohorts (TCGA: *p* < 0.0001, HR = 2.268; ICGC_PACA_AU: *p* = 0.0173, HR = 1.464; GSE62452: *p* = 0.0459, HR = 1.778; GSE57495: *p* = 0.026, HR = 2.019) (Figure 4C, S2C). Figure S2C showed overall survival of patients with low and high Methyscore in the ICGC_PACA_AU, GSE62452, and GSE57495 cohorts. ROC analysis showed predictive capacity for 1–5 years OS (AUCs: TCGA > 0.714; ICGC_PACA_AU 4‐yr = 0.667; GSE62452 3‐yr = 0.753; GSE57495 > 0.69) (Figure 4D, S2D). Figure S2D showed ROC curves and AUC values in the ICGC_PACA_AU, GSE62452, and GSE57495 cohorts. Univariate Cox regression identified Methyscore as a significant risk factor (HR = 2.628, *p* < 0.0001) (Figure 4E). Multivariate analysis confirmed Methyscore as an independent prognostic factor (HR = 2.357, *p* < 0.001) (Figure 4F). A nomogram integrating Methyscore and significant clinical factors was constructed (Figure 4G). Calibration curves showed good agreement between predicted and actual 1, 3, and 5‐year OS (Figure 4H). The nomogram effectively stratified patients into high and low‐risk groups (Figure 4I). DCA demonstrated superior clinical utility of the nomogram (Figure 4J).

### 3.4 Analysis of the Correlation Between m6A/m1A/m5C‐Related Subtypes and Models With Immune Response

#### 3.4.1 Molecular Subtypes

**FIGURE 5:**
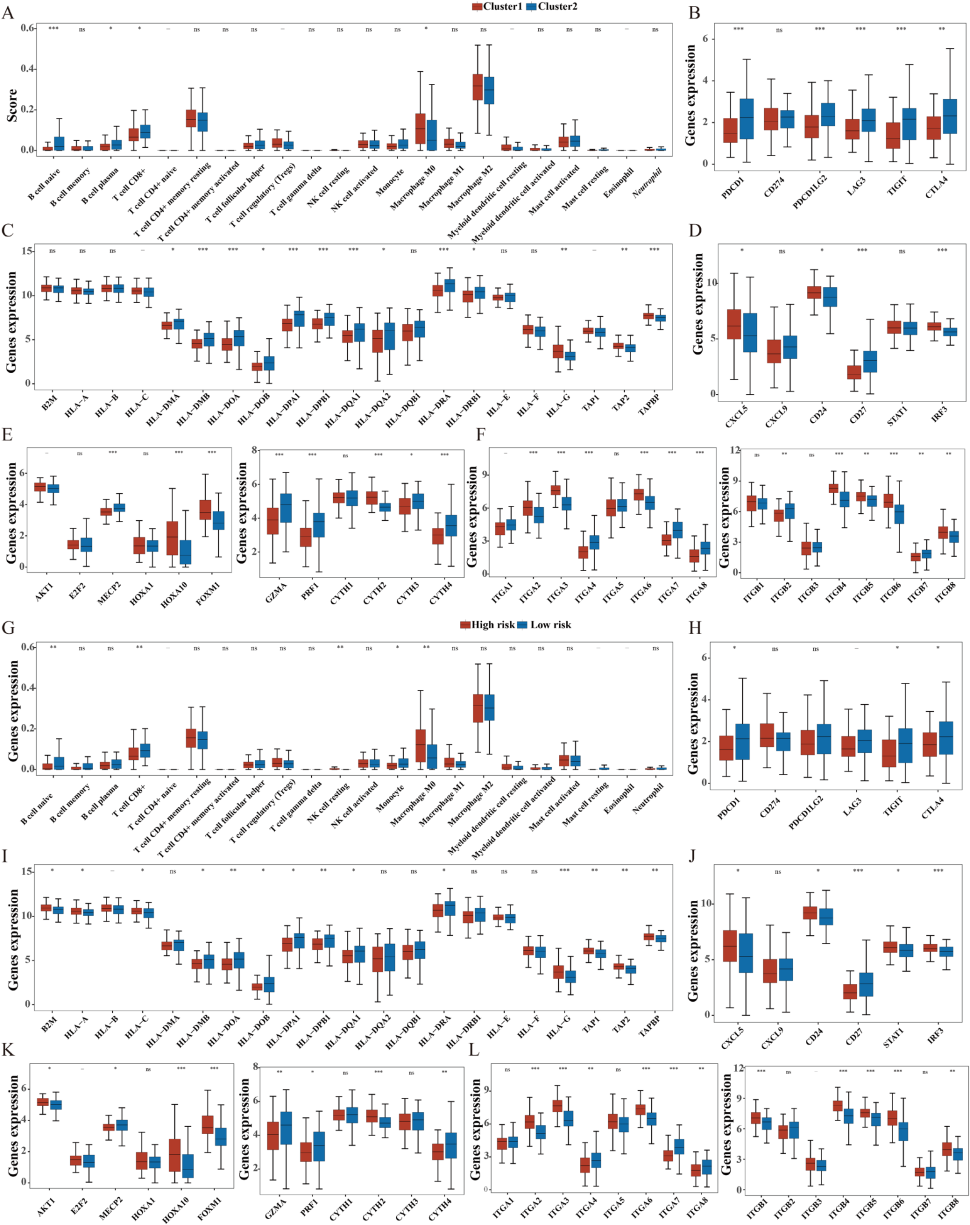
(A)‐(F) The immune landscape of Cluster 1 and Cluster 2 subtypes. (A) Box plots showing the comparison of cellular composition fractions of B cells, CD8+ T cells, CD4+ T cells, helper T cells, regulatory T cells, activated natural killer (NK) cells, M0 macrophages, M1 macrophages, M2 macrophages, monocytes, mast cells, and activated dendritic cells among different subtypes. (B) Box plots displaying the comparison of expressions of PD‐1, PD‐L1, PD‐L2, LAG3, TIGIT, and CTLA4 between C1 and C2 subtypes. (C) Box plots illustrating the comparison of expressions of HLA‐A, HLA‐B, HLA‐C, HLA‐E, TAP1, and B2M between C1 and C2 subtypes. (D) Box plots comparing the expressions of CCL5, CXCL9, CD24, CD27, STAT1, and IRF3 between C1 and C2 subtypes. (E) Box plots comparing the expressions of AKT1, E2F2, MECP2, HOXA1, HOXA10, FOXM1, GZMA, PRF1, CYTH1, CYTH2, CYTH3, and CYTH4 between C1 and C2 subtypes. (F) Box plot comparing the expressions of ITGA and ITGB family genes between C1 and C2 subtypes. (G)‐(L) The immune landscape of high‐risk and low‐risk groups. (G) Box plots showing the comparison of cellular composition fractions of B cells, CD8+ T cells, CD4+ T cells, helper T cells, regulatory T cells, activated NK cells, M0 macrophages, M1 macrophages, M2 macrophages, monocytes, mast cells, and activated dendritic cells between different risk groups; (H) Box plots showing the comparison of expression levels of PD‐1, PD‐L1, PD‐L2, LAG3, TIGIT, and CTLA4 between different risk groups; (I) Box plots depicting the comparison of expression levels of HLA‐A, HLA‐B, HLA‐C, HLA‐E, TAP1, and B2M between high‐risk and low‐risk groups; (J) Box plots comparing the expression levels of CCL5, CXCL9, CD24, CD27, STAT1, and IRF3 between different risk groups; (K) Box plots comparing the expression levels of AKT1, E2F2, MECP2, HOXA1, HOXA10, FOXM1, GZMA, PRF1, CYTH1, CYTH2, CYTH3, and CYTH4 between different risk groups; (L) Box plot comparing the expressions of ITGA and ITGB family genes between low‐risk and high‐risk groups.

We cautiously explored the immune landscape of these subtypes. CIBERSORT revealed distinct immune cell infiltration patterns between Clusters 1 and 2 (Figure 5A). Cluster 2 exhibited higher expression of T‐cell exhaustion markers (PD‐1, PD‐L1, PD‐L2, LAG3, TIGIT, CTLA4) (Figure 5B), most MHC genes (Figure 5C), but lower expression of CXCL5, CD24, and IRF3 (Figure 5D). Kinase regulator MECP2 (Figure 5E), cytolytic genes (GZMA, CYTH2–4) (Figure 5E), and integrin genes (Figure 5F) also differed significantly.

#### 3.4.2 Risk Model Groups

Similarly, immune infiltration differed between high and low‐risk groups (Figure 5G). The low‐risk group had higher expression of immune checkpoints (Figure 5H), antigen presentation genes (Figure 5I), CD27, but lower expression of CXCL5, CD24, IRF3, STAT1 (Figure 5J). Kinase regulators (AKT1, HOXA10, FOXM1), cytolytic genes (CYTH1‐3) (Figure 5K), and integrin genes (Figure 5L) showed significant differences as well. However, it is important to note that these molecular differences in m6A/m5C/m1A‐related subtypes and models represent a distinct immune landscape rather than direct evidence of clinical benefit.

### 3.5 Exploratory Analysis of Immunotherapy Potential in Different Subtypes and Risk Models

#### 3.5.1 Molecular Subtypes

**FIGURE 6:**
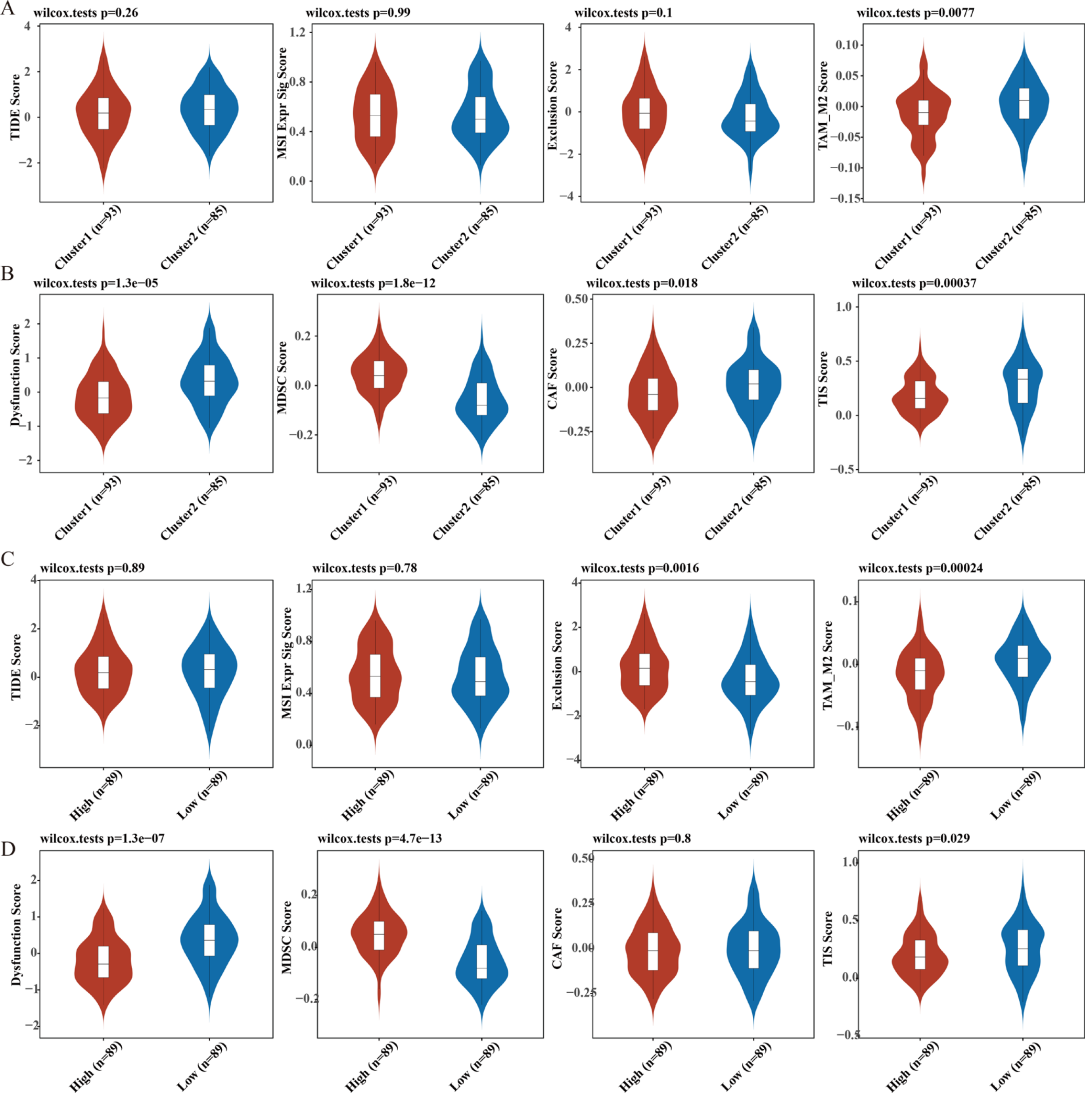
(A) Violin plots illustrate comparisons of TIDE, MSI_Expr_Sig, exclusion, and TAM_M2 values across different C1/C2 subtypes, representing TIDE, MSI, T cell exclusion dysfunction, and TAM scores, respectively. (B) Violin plot compares dysfunction values between different C1/C2 subtypes, representing T cell dysfunction scores, MDSC and CAF scores, and TIS scores, respectively. *p*‐values for comparisons between the two subtypes were calculated using the Wilcoxon test. (C) Violin plots illustrate comparisons of TIDE, MSI_Expr_Sig, exclusion, and TAM_M2 values across different low/high‐risk subtypes, representing TIDE, MSI, T cell exclusion dysfunction, and TAM scores, respectively. (D) Violin plot compares dysfunction values between different low/high‐risk subtypes, representing T cell dysfunction scores, MDSC and CAF scores, and TIS scores, respectively. *p*‐values for comparisons between the two subtypes were calculated using the Wilcoxon test.

We utilized the Tumour Immune Dysfunction and Exclusion (TIDE) algorithm to preliminarily assess the potential for immune evasion. TIDE analysis revealed no significant difference in overall TIDE scores between Clusters 1 and 2, suggesting a similar likelihood of immunotherapy resistance inherent to pancreatic cancer. Cluster 2 exhibited higher TAM_M2 and T‐cell dysfunction scores, along with a higher Tumour Inflammation Signature (TIS) score, whereas Cluster 1 had a higher MDSC score (Figure 6A,B).

#### 3.5.2 Risk Model Groups

Similarly, we applied the TIDE algorithm to the risk model groups. Consistent with the subtype analysis, no significant difference in TIDE scores was found between the high and low‐risk groups. The high‐risk group correlated with higher T‐cell exclusion/dysfunction and MDSC scores, while the low‐risk group had a significantly higher TIS score (Figure 6D). Altogether, these computational predictions imply that the identified risk groups and subtypes possess distinct immune‐suppressive features but do not necessarily translate to a differential clinical response to immunotherapy in the context of pancreatic cancer.

### 3.6 The Biological Function of TRMT61B in PDAC

**FIGURE 7:**
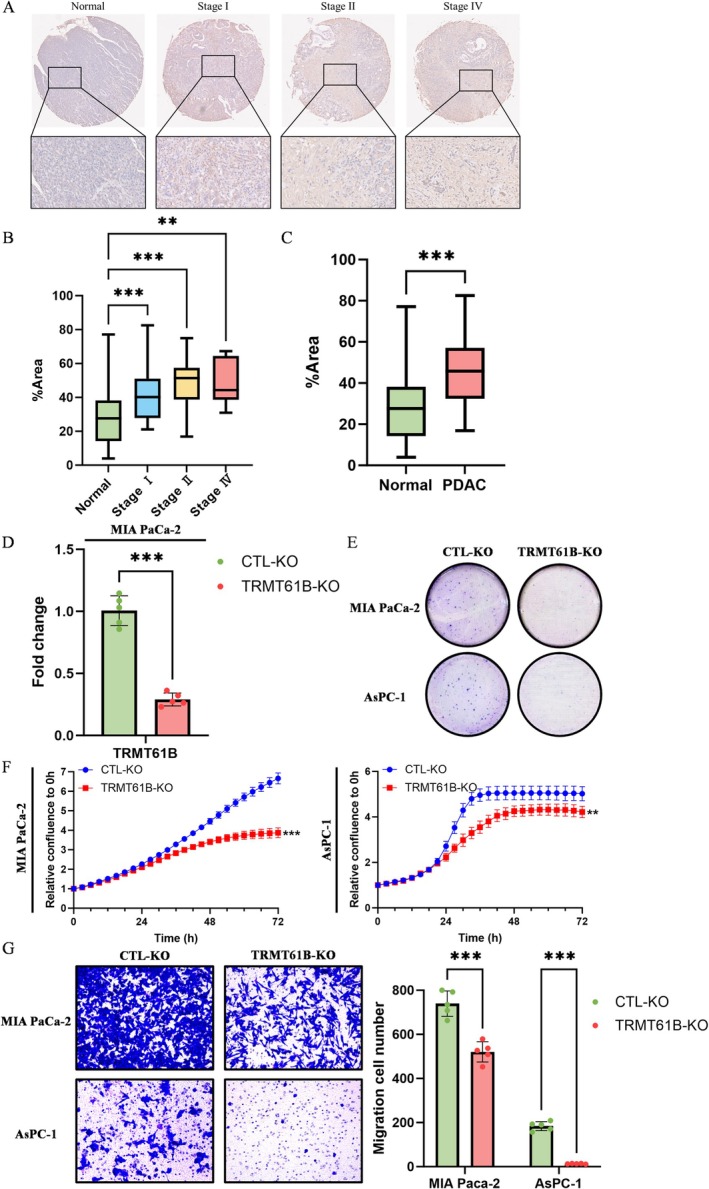
The biological function of TRMT61B in PDAC. (A) Representative IHC images showing TRMT61B expression in normal tissues and pancreatic cancer tissues at different stages (magnification: 5 × and 40 × ). (B) Comparison of TRMT61B expression levels in normal tissues versus pancreatic cancer tissues at different stages. (C) Overall expression difference of TRMT61B between normal and pancreatic cancer tissues. (D) TRMT61B was knocked down using lentivirus, and the knockdown efficiency was verified by qPCR. (E) Cell proliferative capacity was assessed by colony formation assay after TRMT61B knockdown. (F) Cell proliferative capacity was examined by Incucyte proliferation assay after TRMT61B knockdown. (G) Cell invasive ability was determined by Transwell invasion assay after TRMT61B knockdown.

To validate the aforementioned findings, we conducted molecular and cellular experiments and pinpointed TRMT61B among the nine genes derived from our LASSO‐based prognostic model, subsequently investigating its functional role in pancreatic cancer. TRMT61B encodes a mitochondrial tRNA methyltransferase that catalyses site‐specific nucleotide modifications indispensable for mitochondrial translation and bioenergetic function. Immunohistochemical analysis of a pancreatic cancer tissue microarray (TMA) containing 150 samples showed the expression of TRMT61B in normal tissues and pancreatic cancer tissues at different stages, as depicted in Figure 7A. The results revealed that the expression of TRMT61B was significantly higher in all stages of pancreatic cancer compared to normal tissues (Figure 7B), and the expression level in pancreatic cancer tissues was also markedly upregulated (Figure 7C). These findings corroborate previous analyses and provide further evidence suggesting a potential oncogenic role for TRMT61B in pancreatic cancer. In addition, we performed loss‐of‐function studies in AsPC‐1 and MIA PaCa‐2 cells (Figure 7D). Subsequent functional assays, including colony formation, Incucyte proliferation, and Transwell invasion experiments, demonstrated that TRMT61B knockdown significantly suppressed colony formation (Figure 7E), cell proliferation (Figure 7F), and invasion (Figure 7G).

### 3.7 The Expression Patterns of TRMT61B in Pancreatic Cancer Cells and Immune Microenvironment

**FIGURE 8:**
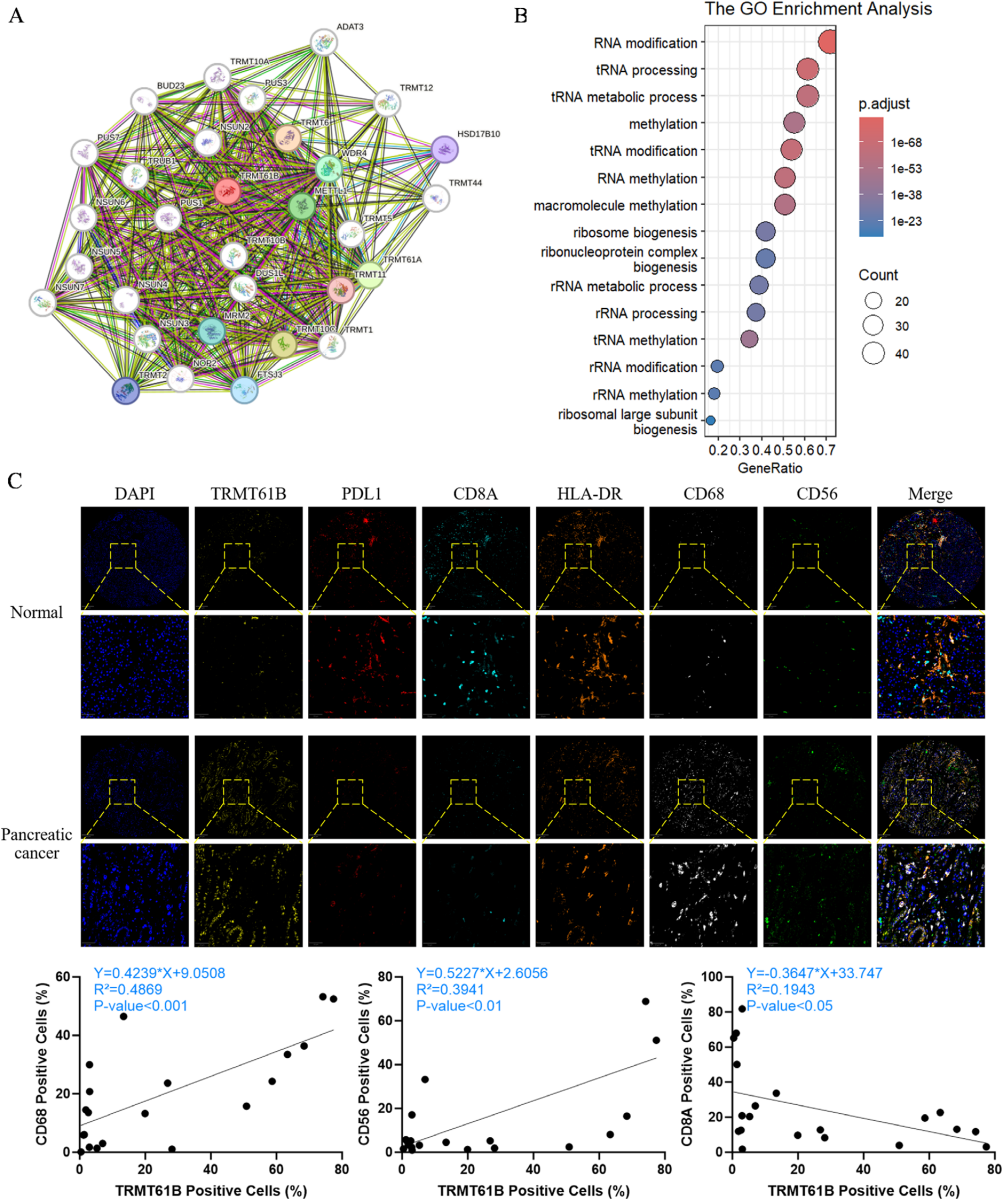
The expression patterns of TRMT61B in pancreatic cancer cells and immune microenvironment. (A) The functional protein association network of TRMT61B from the STRING database (string‐db.org). (B) GO enrichment analysis of TRMT61B gene. (C) Multicolor immunofluorescence analysis in normal and pancreatic cancer tissues. TRMT61B is labelled in yellow. Meanwhile, PDL1 (red), CD8A (cyan), HLA‐DR (orange), CD68 (white), CD56 (green), DAPI (blue) labelled immune checkpoint, cytotoxic T lymphocytes, antigen‐presenting cells, macrophages, natural killer cells and nuclei, respectively. The correlation of TRMT61B positive cells and immune cells is quantified.

To investigate the interaction network and expression patterns of TRMT61B within the immune microenvironment, we performed functional protein association network analysis, GO enrichment analysis, and multicolor immunofluorescence (mIF) staining (Figure 8). The protein association network revealed that TRMT61B is functionally linked to several RNA modification enzymes, including NSUN2, PUS1, TRMT10B, and METTL1 (Figure 8A). Furthermore, GO enrichment analysis indicated that TRMT61B is significantly involved in RNA modification, tRNA processing, and tRNA metabolic processes (Figure 8B). Multicolor immunofluorescence staining was performed, followed by systematic quantitative image analysis. Using QuPath‐0.6.0 software, we quantified two key parameters: (i) the proportion of TRMT61B‐positive cells in tumour tissues and normal tissues; (ii) the proportion of various immune cells in tumour tissues and normal tissues. Two‐way analysis of variance (ANOVA) was used for statistical comparisons between tumour tissues and normal tissues. Pearson correlation analysis and simple linear regression analysis were performed to evaluate the quantitative association between TRMT61B expression level and the levels of various immune cells. The results (Figure 8C) showed that in comparison to normal tissues, tumour tissues displayed higher TRMT61B, but lower PD‐L1 and HLA‐DR expression. In addition, the expression of CD56 and CD68 were higher in pancreatic cancer tissues than normal tissues, while CD8A showed lower expression. Furthermore, the proportion of TRMT61B‐positive cells was positively correlated with the expression levels of CD56 and CD68, and negatively correlated with the expression level of CD8A. These results revealed that pancreatic tissues had more macrophages and NK cells but less CD8+ T cells and APCs, correlating with the trend of cluster 1 and the high‐risk group mentioned earlier, which further proved the oncogenic effect of TRMT61B.

## 4 Discussion

This study utilized the TCGA‐PAAD cohort and three external datasets (GSE62452, GSE57495, and ICGC_PACA_AU) for analysis. Firstly, we screened 43 m^6^A/m^1^A/m^5^C modification‐related genes and analysed their expression differences between tumour and normal tissues. The analysis results showed that these modification‐related genes exhibited significant expression differences among different tissues. Further, by constructing a protein–protein interaction network using the String database, we found a high co‐expression relationship and a strong interaction network among m^6^A/m^1^A/m^5^C‐related genes. Next, we divided pancreatic cancer patients into two subtypes through consensus clustering analysis and discovered significant survival differences between the subtypes. To construct a prognostic prediction model, we used LASSO regression to screen 9 m^6^A/m^1^A/m^5^C‐related genes associated with prognosis and built a risk scoring model. We also constructed a nomogram model that combined clinical characteristics and risk scores, further improving the accuracy of prognostic prediction. This model can assist clinicians in better assessing the prognosis of pancreatic cancer patients and provide guidance for personalised treatment. By evaluating immune cell infiltration, we characterised the distinct immune landscapes across different subtypes and risk models. However, given that pancreatic cancer is an immunologically cold tumour, these observed immune features should be viewed as descriptive phenotypes of the tumour microenvironment rather than direct evidence for identifying actionable immunotherapy targets. Meanwhile, the predictive model based on LASSO regression showed that the TRMT61B gene was relatively highly expressed in both high and low‐risk groups, suggesting it may play a pro‐tumorigenic role. These findings have laid an important theoretical foundation for subsequent experimental research. In the future, we still need to conduct more research to further validate the clinical application value of the model and explore the specific mechanisms of m^6^A/m^1^A/m^5^C‐related genes in the development of pancreatic cancer.

It can be found from previous studies that over 100 different types of RNA modification have been identified. Methylation of different RNA species has emerged as a critical regulator of transcript expression [20].

RNA methylation and its related downstream signalling pathways are involved in a plethora of biological processes, including cell differentiation, sex determination, and stress response, and others [21, 22]. Increasing evidence indicates that this process is closely connected to cancer cell proliferation, cellular stress, metastasis, and immune response. The main forms of RNA methylation include N1‐methyladenosine (m1A), N6‐methyladenosine (m6A), 5‐methylcytosine (m5C), N7‐methylguanosine (m7G), and 3‐methylcytidine (m3C), highlighting its widespread presence and importance in shaping the complex landscape of gene regulation [23, 24, 25, 26]. Among them, m5C modification has received extensive attention in recent years. It not only affects the stability, translation, and transport of RNA but is also associated with various types of cancer [3, 10, 27, 28]. TRMT61B, has recently been characterized as a mitochondrial tRNA methyltransferase that also methylates cytosolic mRNAs in a sequence‐specific manner, as one of the m1A methyltransferases, has been found to be associated with tumour homologous mutations, cell proliferation, and survival [29, 30]. In previous studies, TRMT61B has been identified as an oncogene in various tumours, but its role in pancreatic cancer has not been fully discussed [10, 11, 12, 13].

In contrast to previous studies, our study has investigated the close relationship between m^6^A/m^1^A/m^5^C modifications and the prognosis of PAAD patients, and identified 43 differentially expressed genes. Based on the results of consensus clustering analysis, we divided PAAD patients into two subtypes. Meanwhile, using these differentially expressed genes, we constructed a risk scoring model. We validated the robustness of our results using external data. By evaluating immune cell infiltration and immune response signatures, we delineated the immune heterogeneity between subtypes and risk models. It should be noted that while these immune profiles correlate with prognostic differences, they do not inherently imply a causal relationship with immunotherapy efficacy or clinical benefit. The predictive model constructed based on LASSO regression suggests that TRMT61B may play a crucial role in the progression of pancreatic cancer. Subsequent studies demonstrate that TRMT61B is highly expressed in pancreatic cancer and is associated with alterations in immunodepression content, playing a significant role in the development of pancreatic cancer.

However, our study still has some limitations. First, more comprehensive clinical factors should be included to determine whether the risk score is an independent prognostic factor for PAAD. Second, we still need further in vitro and in vivo experiments to investigate the impact of m^6^A/m^5^C/m^1^A‐related genes on the identification of pancreatic cancer molecular subtypes and prognosis prediction. Third, the functional mechanism of TRMT61B in pancreatic cancer is not yet clear. The downstream molecular mechanisms and apoptotic effects of TRMT61B represent an important direction for future research, and experimental validation of these pathways will be pursued in subsequent studies. This work aims to identify potential targets for early diagnosis and prognostic markers. Although we observed distinct immune cell infiltration patterns and differential expression of immune checkpoints between molecular subtypes and risk groups, we must interpret these findings with caution. Pancreatic cancer is characterized by a dense stromal barrier and highly immunosuppressive microenvironment, leading to limited response to current immunotherapies. The lack of available PDAC immunotherapy cohorts precludes direct validation of our immunotherapy response prediction, which constitutes an inherent constraint of the current work. Therefore, the immune signatures identified in this study should be viewed as descriptive molecular features associated with m^6^A/m^1^A/m^5^C modifications. The causal relationship between these epigenetic modifications, immune modulation, and clinical outcome remains to be elucidated in future by further clinical trials.

## Author Contributions

**Mingzhen Wang:** supervision. **Abulaihaiti Tuergong:** resources, data curation. **Yuxuan Guo:** supervision. **Hongrui Liu:** supervision. **Xujun Liu:** resources, supervision. **Wenzhe Si:** conceptualization, investigation, funding acquisition, supervision, resources, project administration, methodology, writing – review and editing. **Xiao Huo:** resources, supervision, data curation, software, formal analysis. **Xia Ting:** resources. **Yunyang Wang:** writing – original draft, methodology, validation, investigation, writing – review and editing, visualization. **Xueqing Hong:** software, formal analysis, writing – original draft, writing – review and editing, data curation, visualization. **Dingge Cao:** supervision, writing – review and editing.

## Funding

This work was supported by Beijing Nova Program, 202604841370, 20230484442 Beijing Natural Science Foundation, QY26072, National Natural Science Foundation of China, 82303063, 82103278 Clinical Medicine Plus X‐Young Scholars Project of Peking University, PKU2026PKULCXQ039, pkustar2026011.

## Conflicts of Interest

The authors declare no conflicts of interest.

## Supporting information


**Figure S1:** Validation of Subtype Classification in External Datasets. (A) Cumulative distribution function plot for the ICGC_PACA_AU dataset, showing the consensus distribution for each *k* value (from 2 to 10). In the ICGC_PACA_AU dataset, when *k* = 2, PAAD patients are classified into two molecular clusters based on the m6A/m1A/m5C‐related gene profile. (B) Kaplan–Meier analysis in the ICGC_PACA_AU dataset for the prognosis of PAAD patients belonging to two different molecular clusters. (C) Cumulative distribution function plot for the GSE62452 dataset, showing the consensus distribution for each *k* value (from 2 to 10). In the GSE62452 dataset, when *k* = 2, PAAD patients are classified into two molecular clusters based on the m6A/m1A/m5C‐related gene profile. (D) Kaplan–Meier analysis in the GSE62452 dataset for the prognosis of PAAD patients belonging to two different molecular clusters. (E) Cumulative distribution function plot for the GSE57495 dataset, showing the consensus distribution for each *k* value (from 2 to 10). In the GSE57495 dataset, when *k* = 2, PAAD patients are classified into two molecular clusters based on the m6A/m1A/m5C‐related gene profile. (F) Kaplan–Meier analysis in the GSE57495 dataset for the prognosis of PAAD patients belonging to two different molecular clusters.
**Figure S2:** External Validation for the Gene Signature Prediction Model and Establishment and Evaluation of the Nomogram Survival Model. (A) Distribution of Methyscore adjusted for survival status and time in the ICGC_PACA_AU, GSE62452, and GSE57495 cohorts; (B) Principal Component Analysis (PCA) plot based on Methyscore in the ICGC_PACA_AU, GSE62452, and GSE57495 cohorts; (C) Overall survival of patients with low and high Methyscore (D) ROC curves and AUC values in the ICGC_PACA_AU, GSE62452, and GSE57495 cohorts.

## Data Availability

The data that support the findings of this study are available from the corresponding author upon reasonable request.
