# Supplementary material for: Molecular Subtype Identification and Prognostic Prediction of Pancreatic Cancer Based on m6A/m5C/m1A‐Related Genes
Source: J Cell Mol Med. 2026 Jun 28;30(12):e71251. doi: 10.1111/jcmm.71251 (PMC13311301; doi:10.1111/jcmm.71251)
Supplement: Supplementary file 1 — Figure S1: Validation of Subtype Classification in External Datasets. (A) Cumulative distribution function plot for the ICGC_PACA_AU dataset, showing the consensus distribution for each k value (from 2 to 10). In the ICGC_PACA_AU dataset, when k = 2, PAAD patients are classified into two molecular clusters based on the m6A/m1A/m5C‐related gene profile. (B) Kaplan–Meier analysis in the ICGC_PACA_AU dataset for the prognosis of PAAD patients belonging to two different molecular clusters. (C) Cumulative distribution function plot for the GSE62452 dataset, showing the consensus distribution for each k value (from 2 to 10). In the GSE62452 dataset, when k = 2, PAAD patients are classified into two molecular clusters based on the m6A/m1A/m5C‐related gene profile. (D) Kaplan–Meier analysis in the GSE62452 dataset for the prognosis of PAAD patients belonging to two different molecular clusters. (E) Cumulative distribution function plot for the GSE57495 dataset, showing the consensus distribution for each k value (from 2 to 10). In the GSE57495 dataset, when k = 2, PAAD patients are classified into two molecular clusters based on the m6A/m1A/m5C‐related gene profile. (F) Kaplan–Meier analysis in the GSE57495 dataset for the prognosis of PAAD patients belonging to two different molecular clusters. Figure S2: External Validation for the Gene Signature Prediction Model and Establishment and Evaluation of the Nomogram Survival Model. (A) Distribution of Methyscore adjusted for survival status and time in the ICGC_PACA_AU, GSE62452, and GSE57495 cohorts; (B) Principal Component Analysis (PCA) plot based on Methyscore in the ICGC_PACA_AU, GSE62452, and GSE57495 cohorts; (C) Overall survival of patients with low and high Methyscore (D) ROC curves and AUC values in the ICGC_PACA_AU, GSE62452, and GSE57495 cohorts. [file JCMM-30-e71251-s001.docx]

**Supplementary figures**

**
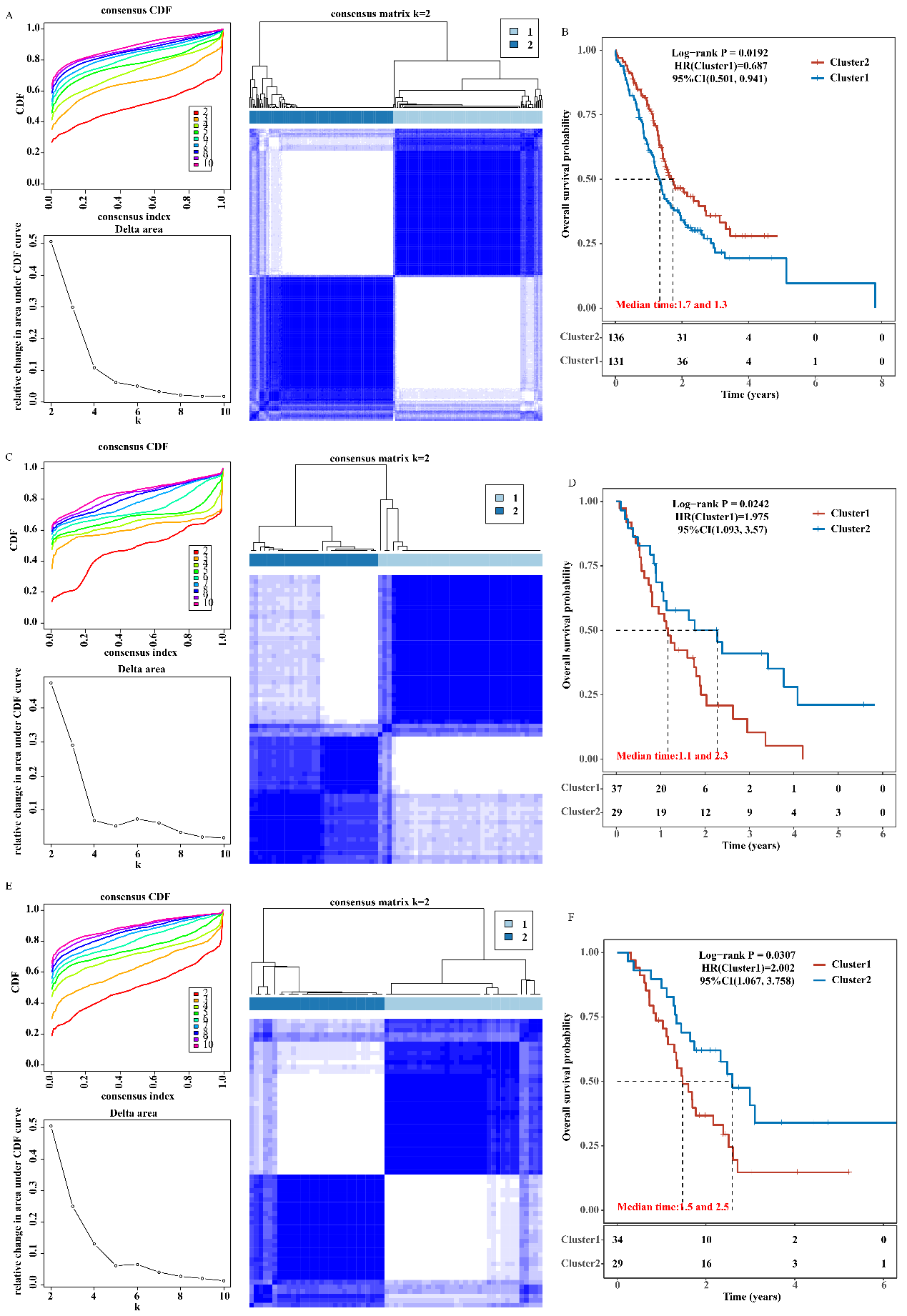
**

Fig. S1 Validation of Subtype Classification in External Datasets. (A) Cumulative distribution function plot for the ICGC_PACA_AU dataset, showing the consensus distribution for each k value (from 2 to 10). In the ICGC_PACA_AU dataset, when k = 2, PAAD patients are classified into two molecular clusters based on the m6A/m1A/m5C-related gene profile. (B) Kaplan-Meier analysis in the ICGC_PACA_AU dataset for the prognosis of PAAD patients belonging to two different molecular clusters. (C) Cumulative distribution function plot for the GSE62452 dataset, showing the consensus distribution for each k value (from 2 to 10). In the GSE62452 dataset, when k = 2, PAAD patients are classified into two molecular clusters based on the m6A/m1A/m5C-related gene profile. (D) Kaplan-Meier analysis in the GSE62452 dataset for the prognosis of PAAD patients belonging to two different molecular clusters. (E) Cumulative distribution function plot for the GSE57495 dataset, showing the consensus distribution for each k value (from 2 to 10). In the GSE57495 dataset, when k = 2, PAAD patients are classified into two molecular clusters based on the m6A/m1A/m5C-related gene profile. (F) Kaplan-Meier analysis in the GSE57495 dataset for the prognosis of PAAD patients belonging to two different molecular clusters.

**
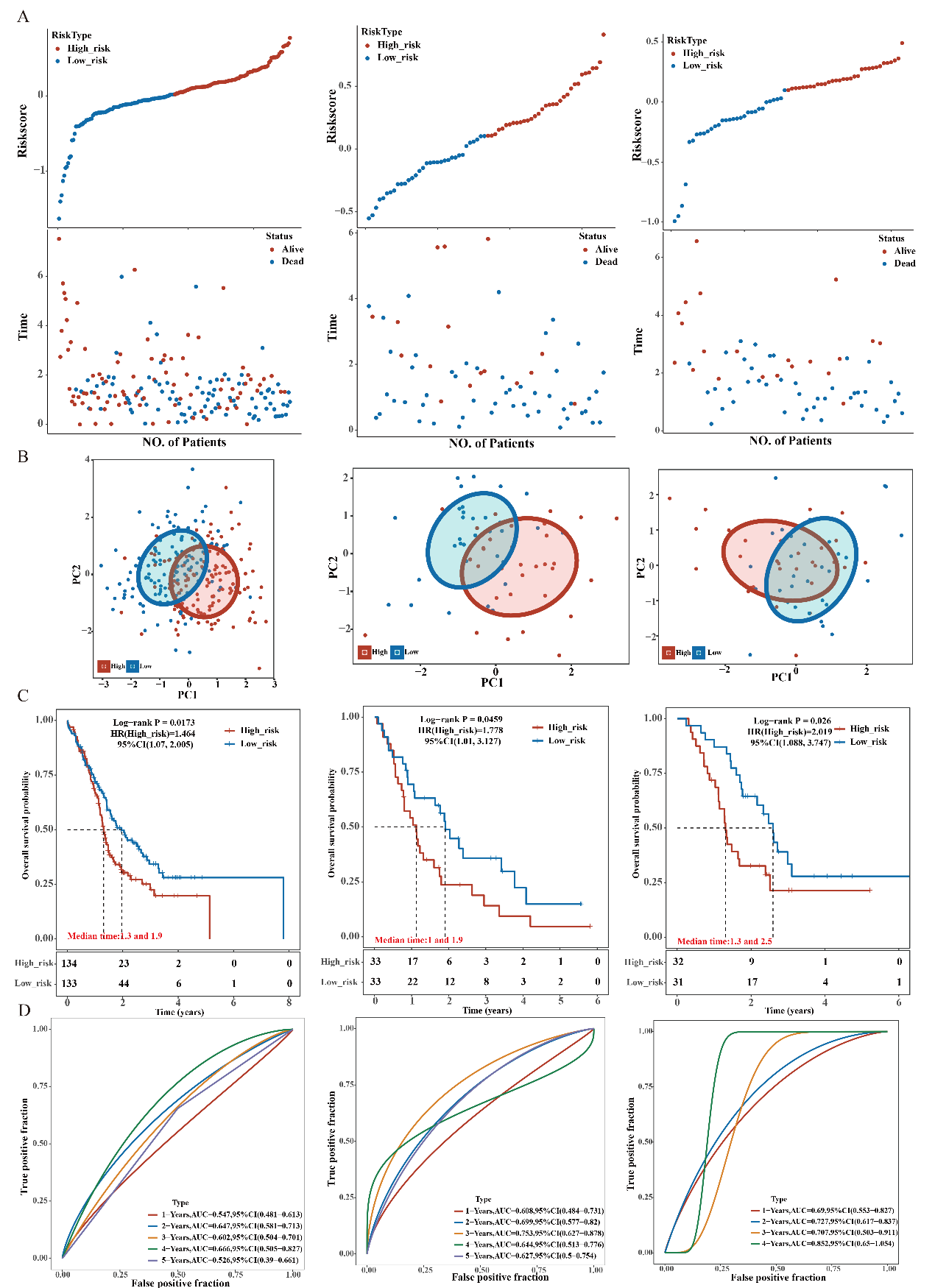
**

Fig. S2 External Validation for the Gene Signature Prediction Model and Establishment and Evaluation of the Nomogram Survival Model. (A) Distribution of Methyscore adjusted for survival status and time in the ICGC_PACA_AU, GSE62452, and GSE57495 cohorts; (B) Principal Component Analysis (PCA) plot based on Methyscore in the ICGC_PACA_AU, GSE62452, and GSE57495 cohorts; (C) Overall survival of patients with low and high Methyscore (D) ROC curves and AUC values in the ICGC_PACA_AU, GSE62452, and GSE57495 cohorts.
